# A Simple 3-Parameter Model for Examining Adaptation in Speech and Voice Production

**DOI:** 10.3389/fpsyg.2019.02995

**Published:** 2020-01-21

**Authors:** Elaine Kearney, Alfonso Nieto-Castañón, Hasini R. Weerathunge, Riccardo Falsini, Ayoub Daliri, Defne Abur, Kirrie J. Ballard, Soo-Eun Chang, Sara-Ching Chao, Elizabeth S. Heller Murray, Terri L. Scott, Frank H. Guenther

**Affiliations:** ^1^Department of Speech, Language, and Hearing Sciences, Boston University, Boston, MA, United States; ^2^Department of Biomedical Engineering, Boston University, Boston, MA, United States; ^3^Department of Speech and Hearing Science, Arizona State University, Tempe, AZ, United States; ^4^Faculty of Health Sciences, The University of Sydney, Sydney, NSW, Australia; ^5^Department of Psychiatry, University of Michigan, Ann Arbor, MI, United States; ^6^Cognitive Imaging Research Center, Department of Radiology, Michigan State University, East Lansing, MI, United States; ^7^Graduate Program for Neuroscience, Boston University, Boston, MA, United States; ^8^The Picower Institute for Learning and Memory, Massachusetts Institute of Technology, Cambridge, MA, United States; ^9^Athinoula A. Martinos Center for Biomedical Imaging, Massachusetts General Hospital, Charlestown, MA, United States

**Keywords:** computational modeling, sensorimotor adaptation, motor control, speech production, voice, auditory feedback

## Abstract

Sensorimotor adaptation experiments are commonly used to examine motor learning behavior and to uncover information about the underlying control mechanisms of many motor behaviors, including speech production. In the speech and voice domains, aspects of the acoustic signal are shifted/perturbed over time via auditory feedback manipulations. In response, speakers alter their production in the opposite direction of the shift so that their perceived production is closer to what they intended. This process relies on a combination of feedback and feedforward control mechanisms that are difficult to disentangle. The current study describes and tests a simple 3-parameter mathematical model that quantifies the relative contribution of feedback and feedforward control mechanisms to sensorimotor adaptation. The model is a simplified version of the DIVA model, an adaptive neural network model of speech motor control. The three fitting parameters of *SimpleDIVA* are associated with the three key subsystems involved in speech motor control, namely auditory feedback control, somatosensory feedback control, and feedforward control. The model is tested through computer simulations that identify optimal model fits to six existing sensorimotor adaptation datasets. We show its utility in (1) interpreting the results of adaptation experiments involving the first and second formant frequencies as well as fundamental frequency; (2) assessing the effects of masking noise in adaptation paradigms; (3) fitting more than one perturbation dimension simultaneously; (4) examining sensorimotor adaptation at different timepoints in the production signal; and (5) quantitatively predicting responses in one experiment using parameters derived from another experiment. The model simulations produce excellent fits to real data across different types of perturbations and experimental paradigms (mean correlation between data and model fits across all six studies = 0.95 ± 0.02). The model parameters provide a mechanistic explanation for the behavioral responses to the adaptation paradigm that are not readily available from the behavioral responses alone. Overall, SimpleDIVA offers new insights into speech and voice motor control and has the potential to inform future directions of speech rehabilitation research in disordered populations. Simulation software, including an easy-to-use graphical user interface, is publicly available to facilitate the use of the model in future studies.

## Introduction

Sensorimotor adaptation paradigms have become an important experimental approach in studying the neural mechanisms of motor control, including speech production. These paradigms are based on the premise that small, often imperceptible, manipulations of sensory feedback result in lasting changes within the sensorimotor system (often referred to as *motor learning*) as participants gradually adapt their movements to compensate for the sensory perturbations. Residual compensatory behavior is evident after the manipulation is removed; such after-effects provide clear evidence of the adaptive changes within the motor system.

**FIGURE 1:**
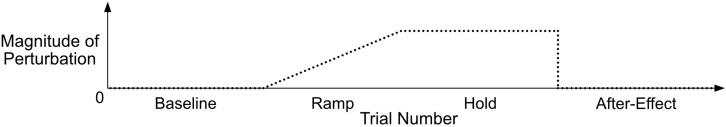
A schematic of a typical sensorimotor adaptation paradigm with four phases. During the *baseline* phase, participants receive normal auditory feedback (magnitude of perturbation = 0). The perturbation is gradually increased from 0 to its maximum value during the *ramp* phase. The maximum perturbation is held constant during the *hold* phase. Auditory feedback immediately returns to normal during the *after-effect* phase.

A typical sensorimotor adaptation paradigm consists of four phases, shown in Figure 1. The paradigm begins with a *baseline* phase^1^ where participants produce stimuli (e.g., syllables, sustained vowels) and receive normal, unaltered auditory feedback. The second phase, referred to as a *ramp* phase, is characterized by a gradual addition of the auditory feedback perturbation. The perturbation is implemented in near real time (typically with a delay of 40 ms or less) using a digital signal processing system and/or personal computer-based software (e.g., Audapter; Cai et al., 2008) and is increased linearly until reaching the maximum perturbation magnitude. The maximum perturbation remains constant during the *hold* phase. The final phase is the *after-effect* phase, where auditory feedback immediately returns to normal. The number of trials per phase varies by study but is often in the range of 10 to 100 trials, with the largest number of trials usually occurring in the hold phase. In addition, the ramp may be more or less gradual (or omitted), and masking noise played on short blocks of trials during the hold phase can be used to assess adaptation in place of the after-effect phase.

Originally adapted from studies of limb motor control, the sensorimotor adaptation paradigm was first applied to formant frequencies during speech by Houde and Jordan (1998). Formant frequencies are peaks in the acoustic spectrum that are related to the overall shape of the vocal tract and are important for differentiating speech sounds. Roughly speaking, the first formant (F1) is inversely related to tongue height (i.e., sounds with higher tongue positions have lower F1 values) whereas the second formant (F2) is related to the location of the tongue constriction along the vocal tract (i.e., sounds with constrictions closer to the lips have higher F2 values). In the study by Houde and Jordan (1998), participants produced CVC syllables containing the vowel/ε/while the first two formants were shifted either toward the vowel/i/or the vowel/a/. Compensation was observed in the opposite direction to the perturbation. During the hold phase, adaptation was assessed by randomly interspersing trials with masking noise so that auditory feedback was unavailable to participants. The masked trials also showed evidence of compensatory behavior, revealing adaptation within the speech motor system to the formant perturbations.

Since the first application of the sensorimotor adaptation paradigm to speech, a number of adaptation studies have supported the original findings for formant perturbations (e.g., Purcell and Munhall, 2006; Villacorta et al., 2007) as well as several additional acoustic manipulations, including shifting the center of spectral energy of fricatives (Shiller et al., 2007, 2009) and perturbing fundamental frequency (*f*_o_, the acoustic correlate of pitch) during sustained phonation (Jones and Munhall, 2000; Hawco and Jones, 2010). The findings have also been generalized to perturbations of pitch and formant frequencies in Mandarin, a tonal language (Jones and Munhall, 2002, 2005; Cai et al., 2010), and to sentence-level stimuli with formants of multiple vowels perturbed within an utterance (Lametti et al., 2018). Keough et al. (2013) demonstrated that the presence or absence of specific instructions to attend to the acoustic manipulations does not affect adaptation suggesting that adaptation is under automatic rather than conscious control. Links have also been demonstrated between perceptual abilities and sensorimotor adaptation. For example, both Villacorta et al. (2007) and Martin et al. (2018) found that speakers who have better auditory acuity showed greater adaptive responses to perturbations of F1, and other researchers have shown that sensorimotor adaptation can result in changes in the speech perception of the adapted speech sound in addition to non-adapted but coarticulatory-dependent speech sounds (Shiller et al., 2009; Lametti et al., 2014; Schuerman et al., 2017).

Most studies of sensorimotor adaptation in speech have involved neurologically normal adult speakers. More recently, the sensorimotor adaptation paradigm has been used to investigate sensorimotor adaptation in children and individuals diagnosed with communication disorders. Evidence of adaptation has been shown in children as young as three; however, the magnitude of the adaptive response is not as great as adults (Scheerer et al., 2016) and adaptation does not appear to have a reliable effect on their perceptual representations (Shiller et al., 2010). In the realm of communication disorders, the paradigm has been used to assess speech motor control of individuals with Parkinson’s disease (PD; Mollaei et al., 2013; Abur et al., 2018), hyperfunctional voice disorder (Stepp et al., 2017), cerebellar degeneration (Parrell et al., 2017), apraxia of speech (Ballard et al., 2018), autism (Demopoulos et al., 2018), developmental dyslexia (van den Bunt et al., 2017), and stuttering (Daliri et al., 2018). The findings of these studies have important implications for uncovering the underlying neural mechanisms of these disorders and may shed light on future treatment strategies.

As the studies reviewed above have demonstrated, the speech sensorimotor adaptation paradigm provides an informative window into learning in the speech motor system. However, it is important to realize that speech output under perturbed auditory feedback is a combination of online sensory feedback control processes (i.e., motor corrections based on sensory errors detected within the ongoing production) and adaptive processes that affect future productions whether or not they are perturbed. This makes it difficult to determine the true level of adaptation (in the sense of trial-to-trial learning) from the experimental data since this adaptive component is “corrupted” by online, within-trial contributions from sensory feedback control.

The widely used Directions Into Velocities of Articulators (DIVA) model of speech production (Guenther, 2006, 2016) proposes that the overall motor command to the speech articulators consists of three main components: (1) an auditory feedback control component that is invoked when errors are detected in auditory feedback, (2) a somatosensory feedback component that is invoked when errors are detected in somatosensory feedback from the speech articulators, and (3) a feedforward component that utilizes stored motor programs for the sounds being produced. Furthermore, the model posits that the feedforward command for future productions is updated based on sensory errors detected in the current trial. This adaptation process has been shown to be capable of accounting for compensatory responses seen in a prior sensorimotor adaptation experiment (Villacorta et al., 2007), though the relative contributions of the three different control processes could not be uniquely determined due to the relatively high number of free parameters in the full DIVA model.

Relatively complex models, such as the full DIVA model, are important for expanding our understanding of the neural bases of speech and providing theoretical frameworks to unify findings from a wide range of experimental paradigms. However, they are limited in their usefulness as a tool for characterizing the impaired speech of individuals in the clinic. Specifically, their complexities and parameter redundancies preclude a unique, meaningful model “fit” for the individual. The purpose of the current article is to describe a simple 3-parameter model based on DIVA that can be used to dissociate the contributions of the auditory feedback-based, somatosensory feedback-based, and feedforward control processes in experimentally measured sensorimotor adaptation responses. We will refer to this model as SimpleDIVA throughout the article. The overarching goal of SimpleDIVA is to distil a complex model into its most fundamental components so it can be used to derive a meaningful characterization of function/dysfunction in each of the three main sub-controllers for speech in individuals with speech disorders. The first step in this process is to verify that the model provides adequate fits to existing group datasets. As detailed in the next section, the model’s parameters characterize the gains of the auditory and somatosensory feedback control systems as well as the trial-to-trial adaptation rate of the feedforward control system. Given a sensorimotor adaptation dataset, optimal values of these parameters for fitting the data are derived (i.e., are data-driven); the resulting parameters provide an estimate of the relative roles of the three different control subsystems in the corresponding experiment. For the purposes of the current article, we focus on adaptation experiments involving auditory feedback perturbations, though in principle the same model can be used to analyze the results of adaptation experiments involving somatosensory perturbations applied to the speech articulators (e.g., Tremblay et al., 2003; Nasir and Ostry, 2006) as well as experiments involving perturbations to both auditory and somatosensory feedback (Feng et al., 2011; Lametti et al., 2012).

The remainder of the article is organized as follows. After a description of the SimpleDIVA model, we report a series of 10 simulations in which the model is fit to existing sensorimotor adaptation datasets. Simulations 1 and 2 examine adaptation with perturbations applied to a single auditory dimension (F1). Simulations 3 and 4 assess adaptation with perturbations applied simultaneously to multiple auditory dimensions, specifically F1 and F2. Simulation 5 evaluates adaptation when applying a perturbation to *f*_o_ under two different experimental conditions, first with an upward perturbation and then with a downward perturbation. Simulations 6 and 7 model *f*_o_ adaptation when the measurement of *f*_o_ is captured early as compared to late in the trial. Simulations 8 and 9 model data from an F1 experiment with a gradual perturbation onset condition and fit the resulting parameters to a second experimental condition with a sudden perturbation onset. The final simulation models all included F1 data in a single simulation to derive optimal model parameters for predicting responses to future F1 adaptation studies. We then summarize the contribution of the work to the literature and suggest future directions for using the SimpleDIVA model.

## Materials and Methods

The following equations characterizing the SimpleDIVA model capture the key aspects of the DIVA model in a simplified form that involves only three free parameters that can be adjusted to fit a particular dataset. For the sake of readability, the equations will assume that the adaptation experiment being modeled involves F1, though the same equations apply to other auditory parameters, as illustrated in the simulations described in the next section. We will denote the target value of F1 for the experimental stimuli as F1_*T*_ and define it to be equal to the mean of the F1 values produced by the participant during the baseline portion of the experiment. We assume that F1_*T*_ remains constant over the course of the experiment; i.e., the participant does not change what they consider to be a correct-sounding production.

In effect, SimpleDIVA focuses on the subspace of the high-dimensional motor space that corresponds to changes in F1. This allows us to replace a high-dimensional motor command vector with a single variable corresponding to the effect of that motor command on F1. In this way, the overall motor command to the speech articulators becomes an F1 value that we will call F1_*produced*_. Equation 1 defines F1_*produced*_ on a given trial or block (indexed by n) as:

(1) $$\text{F}\,1_{p\,r\,o\,d\,u\,c\,e\,d}\,\left(n\right)=\text{F}\,1_{F\,F}\,\left(n\right)+\Delta \,\text{F}\,1_{F\,B}\,\left(n\right)$$

Simply stated, the F1 value produced on a trial is a combination of a feedforward command (F1*_*FF*_*) and a sensory feedback-based correction (ΔF1_*FB*_) that kicks in if/when the auditory and somatosensory feedback controllers detect production errors on the current trial. At the start of each simulation (i.e., for *n* = 1), F1_*FF*_ is initialized to F1_*T*_ corresponding to the assumption that participants have previously learned feedforward commands that successfully produce the target value of F1 under normal feedback conditions.

In the full DIVA model, feedback control consists of two components that are summed together: an auditory-feedback-based component and a somatosensory-feedback-based component. The auditory feedback control component is formed by (i) calculating the difference (error) between a multi-dimensional auditory target and the current auditory feedback, (ii) transforming this auditory error into the motor space, and (iii) scaling the result by an auditory feedback control gain factor. Similarly, the somatosensory feedback control component is formed by calculating the difference between a multi-dimensional somatosensory target and the current somatosensory feedback, transforming this somatosensory error into the motor space, and scaling the result by a somatosensory feedback control gain factor. Again, in SimpleDIVA we focus on only the components of the multi-dimensional somatosensory and auditory spaces that correspond to changes in F1, which means that the auditory and somatosensory targets are both equal to F1_*T*_, and the feedback-based correction on a given trial is characterized by the following equation:

(2) $$\Delta \,\text{F}\,1_{F\,B}\,\left(n\right)=\alpha_{A}*\left(\text{F}\,1_{T}-\text{F}\,1_{A\,F}\,\left(n\right)\right)+\alpha_{s}*\left(\text{F}\,1_{T}-\text{F}\,1_{S\,F}\,\left(n\right)\right)$$

where F1_*AF*_ is the value of F1 heard by the participant (including the perturbation, when one is applied) before feedback control mechanisms kick in on that trial (i.e., F1_*AF*_ = F1_*FF*_ + perturbation size) and F1_*SF*_ is the F1 value corresponding to the current somatosensory feedback before feedback control mechanisms kick in. Since no somatosensory feedback perturbations are being considered herein, F1_*SF*_ on a given trial is simply equal to F1_*FF*_ for that trial in the simulations that follow. The free parameters *a*_*A*_ and *a*_*S*_ are the gains of the auditory and somatosensory feedback control subsystems, respectively. When an auditory perturbation is applied, the auditory feedback controller will attempt to compensate for the perturbation. This compensation will be partially counteracted by the somatosensory feedback controller, which is attempting to keep the vocal tract in the normal somatosensory configuration for the sound. Thus, if all else is equal, increasing α_*A*_ will lead to an increase in the compensatory response to an auditory perturbation commanded by the feedback controller, whereas increasing α_*s*_ will lead to a decrease in the compensatory response to an auditory perturbation.

The equation for updating the feedforward command from trial to trial is:

(3) $$\text{F}\,1_{F\,F}\,\left(n+1\right)=\text{F}\,1_{F\,F}\,\left(n\right)+\lambda_{F\,F}*\Delta \,\text{F}\,1_{F\,B}\,\left(n\right)$$

where λ_*FF*_ is a learning rate parameter for the feedforward command. That is, the feedforward command for the next trial is updated by adding some fraction (characterized by λ_*FF*_) of the feedback-based corrective command from the current trial, as in the full DIVA model.

To fit the SimpleDIVA model to a particular dataset, a particle swarm optimization procedure was used to find optimized values of the three free parameters of the model (α_*A*_, α_*S*_, and λ_*FF*_) to fit the mean data for each trial/block in each condition. In this procedure, the system is initialized with a population of 1000 random sets of parameter values (“particles”) and iterated until convergence to obtain an optimized parameter set. In each iteration, all parameter sets are evaluated by computing the root mean square error (RMSE) of their fits to the data, and a fraction of all sets is replaced by random linear combinations of those parameter sets currently producing the best fits. The procedure stops when all 1000 parameter sets converge within a 1% range of the optimal solution or after 100 consecutive iterations without any improvement in the optimal fit to the data. When the procedure stops, the optimal parameter set among the remaining 1000 sets is selected as the solution. Parameter values were limited to the range [0,1] except where noted, in keeping with their mechanistic interpretations in the model^2^. For each model fit, the optimization procedure was run 10 times in order to evaluate any potential residual variability due to initial conditions or local optima. The resulting parameter estimates were highly robust to initial conditions of the swarm procedure (that is, all 10 runs typically converge on the same optimal parameter set), indicative of reaching the global minimum of the RMSE measure. The minimum-RMSE solution across all 10 repetitions was chosen as the optimized parameter set, and Pearson’s *r* was calculated for this solution to characterize fit quality.

The SimpleDIVA model can also be fit to data from multiple datasets. In these cases, RMSE is first calculated for each dataset individually (using the same parameter values for all datasets), and then the individual dataset error measures are summed to obtain the overall error used in the optimization procedure. This has the effect of weighting the datasets equally regardless of the number of trials in each dataset when determining optimal parameter values. Pearson’s *r* is then calculated across all trials in all datasets, with this measure more heavily influenced by datasets with more trials (simulation 10 in the current article).

**FIGURE 2:**
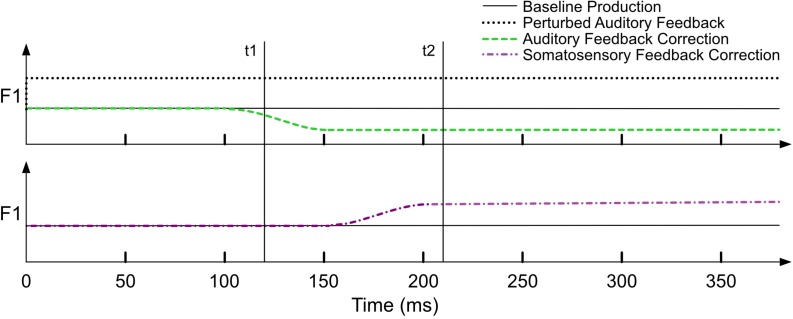
A schematic of the time-course of a single perturbed trial within a sensorimotor adaptation paradigm. The upper panel shows the auditory domain: the auditory perturbation is applied from the beginning of the trial, and the auditory feedback-based correction is evident ∼100 ms post-perturbation onset. The lower panel shows the somatosensory domain: somatosensory feedback-based correction is evident ∼50 ms following auditory feedback-based correction. Across panels, the vertical lines (t1, t2) indicate that the contribution of auditory versus somatosensory feedback control varies depending on when the acoustic measurement of F1 is made within a trial.

An important assumption of the model is that the measurement of F1 in a given trial occurs at a point in time when the auditory feedback controller has already had time to detect and correct for errors, ideally 150 ms or more after perturbed auditory feedback is available to the speaker. This assumption is in place because the model is implicitly expecting contributions from both feedforward and feedback control systems, and it will thus underestimate the influence of feedback control and (consequently) overestimate the amount of trial-to-trial adaptation^3^ if the measurement occurs before feedback control has had time to contribute on the current trial (see simulations 6 and 7). The neural delays associated with sensory feedback processing are approximately 100–150 ms for auditory feedback (Burnett et al., 1997, 1998; Hain et al., 2000) and 20–75 ms for somatosensory feedback (Ludlow et al., 1992; Larson et al., 2008). Figure 2 is a schematic illustration of a hypothetical within-trial time course of a perturbed trial (prior to any adaptation) based on the delays noted above. The auditory perturbation begins with the onset of the trial and remains on for the duration of the trial. An error is detected by the auditory feedback controllers early in the trial and the associated correction is evident starting around 100 ms. This auditory-based correction causes the articulators to change their configuration and, as a result, an error is detected by the somatosensory feedback controller, which begins to correct for the error ∼50 ms later (in the opposite direction of the auditory-based correction). In a typical sensorimotor adaptation experiment, a single measure is taken for each production, typically near the midpoint of a prolonged vowel (e.g., Mollaei et al., 2013; Daliri et al., 2018). If this measurement is taken at 120 ms (t1 in Figure 2), it is likely to underestimate the contribution of feedback control compared to using a later timepoint (e.g., 220 ms, t2). Unless otherwise noted, the studies modeled in this article all involved acoustic measurements that were made more than 150 ms after perturbed auditory feedback was provided.

Across datasets, the optimized model parameters are directly comparable when the experimental and data processing protocols are the same. However, parameters are likely to vary somewhat in response to changes in task, length of utterance, auditory dimension being perturbed, and the timepoint of the acoustic measurement. Random variation associated with recording data from different samples of participants may introduce some degree of uncertainty in the precision of the parameters estimates, but this does not preclude comparisons across datasets assuming the experimental protocols are comparable (see simulations 8 and 9).

## Results

The SimpleDIVA model was used to fit experimental data collected from six prior speech sensorimotor adaptation studies, as detailed in the following subsections. Prior to model fitting, outlier data points greater than two standard deviations from the participant’s mean production in each experimental phase were removed. The mean value of the measured acoustic feature (e.g., F1) across participants was then calculated for each trial in the experiment. All simulations were performed using MATLAB 2018a on a Macintosh computer (macOS Mojave, Version 10.14.3) and replicated on Windows and Linux platforms. Compiled MATLAB code for the SimpleDIVA model is available at http://sites.bu.edu/guentherlab/software/simplediva-app, including a graphical user interface that allows the user to enter new datasets to fit with the model. The graphical user interface is a freely accessible program that does not require a MATLAB license to run.

### Simulation 1: Upward F1 Perturbation

The first simulation involved fitting a dataset was from a classic implementation of the sensorimotor adaptation paradigm as illustrated in Figure 1 that involved an upward perturbation to F1 (Haenchen, 2017). In this study, a group of young healthy speakers of American English (*N* = 18; aged 18–29) produced 60 blocks of trials, with each block including three trials in which the participant produced the word “bed,” “dead,” or “head” in pseudorandom order (180 total individual word trials). For each block, the mean F1 value across the three individual word trials was calculated; this blocked data was used for the model fit. Blocking in this way reduces variability in data plots but has a minimal effect on derived optimal parameters compared to fitting all individual trials. Participants were instructed to say the words slowly and clearly, with an utterance duration between 400 and 600 ms and intensity between 72 and 88 dB SPL. A baseline of 19 blocks (57 individual word trials) was followed by a short ramp phase (1 block) where auditory feedback of F1 was incrementally shifted from 0 to 30% over three trials. The hold and after-effect phases had a further 20 blocks each. Mean F1 was extracted for 60% of the duration of the word, starting from 10% after voice onset time. On average, participants compensated for 31.6% of the perturbation (calculated as change from the baseline to hold phase as a percentage of the maximum perturbation magnitude).

**FIGURE 3:**
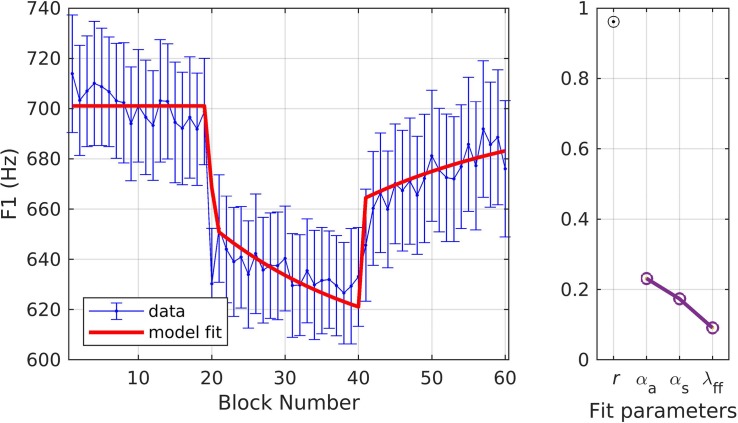
Simulation 1: model fits of a dataset with upward perturbations to F1 (data from Haenchen, 2017; Scott et al., 2019). **(Left)** Mean and standard error of experimental data in blue; model fit in red. **(Right)** Fit quality and optimized parameter values (*r* = correlation coefficient; α_*A*_ = auditory feedback control gain, α_*s*_ = somatosensory feedback control gain, and λ_*FF*_ = learning rate).

Figure 3 shows the model fit to the experimental data. This figure and subsequent figures in this section follow the same format. In the left panel, the mean and standard error of the experimental data are shown in blue and model fits are shown in red. In the right panel, a Pearson’s correlation coefficient (*r*) describes the relationship between the data and model fits, and the parameter estimates are given for α_*A*_, α_*s*_, and λ_*FF*_. The parameter estimates for all 10 optimization runs (see section “Materials and Methods”) are plotted here; however, they typically appear as a single point due to minimal differences between runs, suggesting unique, optimal solutions for these datasets. The reported optimized parameter values and Pearson’s *r* are from the best fit obtained from the 10 optimization runs. The model provided an excellent fit to the data (*r* = 0.96), falling within the standard error of the sample mean for all but one block (the ramp block). Optimized values for the three model parameters (model interpretation given in parentheses) were α_*A*_ = 0.23 (indicating an auditory feedback control gain in which 23% of the detected auditory error for a trial was corrected within that trial), α_*s*_ = 0.17 (indicating a somatosensory feedback control gain in which 17% of the detected somatosensory error for a trial was corrected within that trial), and λ_*FF*_ = 0.09 (indicating a feedforward command learning/update rate in which 9% of the correction from one trial was added to the feedforward command for the next trial).

### Simulation 2: Upward F1 Perturbation With Noise-Masked Trials

The second dataset was from a study involving an upward perturbation to F1 in a group of young healthy speakers of Australian (*N* = 9) and Canadian (*N* = 1) English (mean age = 25.3 ± 3.74 years) (see Supplementary Material, Ballard et al., 2018). A key difference in this experimental paradigm was the use of masking noise to block auditory feedback on certain trials as a way to gauge adaptation in the absence of online auditory feedback-based corrections. On each trial, participants said the word “pear,” “bear,” “care,” or “dare,” pseudorandomly distributed. Productions of “paw” were also recorded but not perturbed and were therefore not included in the current simulation. Participants were instructed to say the words with a clear voice quality, minimal pitch variation, constant speaking volume, and to prolong the vowel for approximately 500 ms. An initial baseline phase consisted of 40 trials with masking noise randomly played on half of the trials, followed by an additional 33 baseline trials with normal auditory feedback. No blocking of trials was performed due to the uneven distribution of noise-masked trials over the course of the experiment. A linear increase in F1 was applied over 59 ramp trials up to the maximum perturbation of 30%. During the hold phase, the maximum perturbation was maintained for a total of 115 trials. After every 15 hold trials, masking noise was played for the following 10 trials. The after-effect phase consisted of 40 noise-masked trials. F1 trajectories were extracted and averaged over the duration of the vowel. Average compensation was 36.5% during the hold phase on trials with unmasked auditory feedback.

**FIGURE 4:**
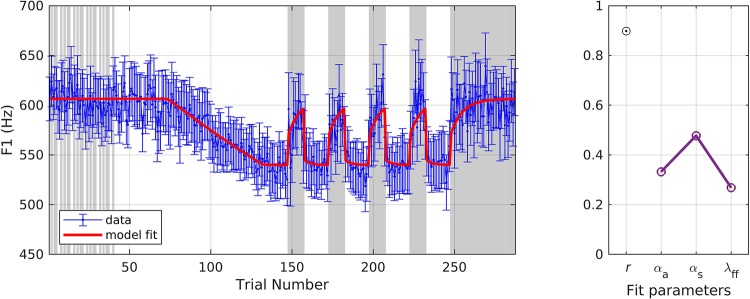
Simulation 2: model fits of a dataset with upward perturbations in F1 and noise-masked trials (indicated with gray shading) interspersed during baseline and hold phases (data from Ballard et al., 2018). **(Left)** Mean and standard error of experimental data in blue; model fit in red. **(Right)** Fit quality and optimized parameter values (*r* = correlation coefficient; α_*A*_ = auditory feedback control gain, α_*s*_ = somatosensory feedback control gain, and λ_*FF*_ = learning rate).

As shown in Figure 4, the model again provided an excellent fit to the data (*r* = 0.90), and the model fits fell within the standard error of the data on 273/287 (95.1%) of trials, including both unmasked and masked trials. The optimized parameter values were α_*A*_ = 0.33 (higher than in simulation 1, indicating a higher compensatory response to the perturbation), α_*s*_ = 0.48 (higher than in simulation 1, indicating more resistance to compensatory responses that moved the production away from its normal state), and λ_*FF*_ = 0.27 (a bit higher than in simulation 1, indicating more adaptation of the feedforward command). Further, the higher α_*s*_ value compared to α_*A*_ indicates that, according to the model, the somatosensory feedback controller has a larger influence than the auditory feedback controller in this experimental protocol compared to simulation 1.

An interesting aspect of this simulation is the fact that the model captures a characteristic of the masked trials during the hold phase that was somewhat unexpected. At first glance, one might expect the F1 values produced during a sequence of consecutive noise-masked trials to remain steady since no auditory perturbation is detected. Instead, as captured by the model, there is a tendency for F1 to increase gradually during such noise-masked sequences. This occurs in the model because somatosensory feedback control remains active during the noise-masked trials, and the somatosensory feedback control system attempts to move the production closer to the normal (pre-perturbation) configuration, in effect counteracting the compensatory adaptation that occurs during unmasked trials in the hold phase.

### Simulations 3 and 4: F1 and F2 Perturbed Simultaneously

Simulations 3 and 4 provide fits to data from an experiment in which young healthy American English speakers (*N* = 14; mean age = 23.7 ± 6.92 years) underwent an adaptation paradigm in which both F1 and F2 were perturbed simultaneously (Daliri et al., 2018; data from only the adult non-stuttering group included here). The experiment involved a total of 90 trials; 18 baseline, 18 ramp, 36 hold, and 18 after-effect trials. The target words were “bed,” “Ted,” and “head,” randomized within each block of three trials, and participants were instructed to produce word durations between 300 and 700 ms and intensities between 72 and 88 dB SPL. The ramp phase was characterized by a gradual increase in F1 to a max perturbation of 25% and a gradual decrease in F2 to a max perturbation of −12.5%. The other three phases followed the standard paradigm. F1 and F2 trajectories were extracted using a custom-written MATLAB script. Mean F1 and F2 were estimated at the center of the vowel (40–60% of the vowel duration) and blocked data (mean of every three trials) were used for model fitting. In response to the F1 perturbation, participants compensated by an average of 21.3%, whereas for the F2 perturbation, they compensated by 3.87%.

**FIGURE 5:**
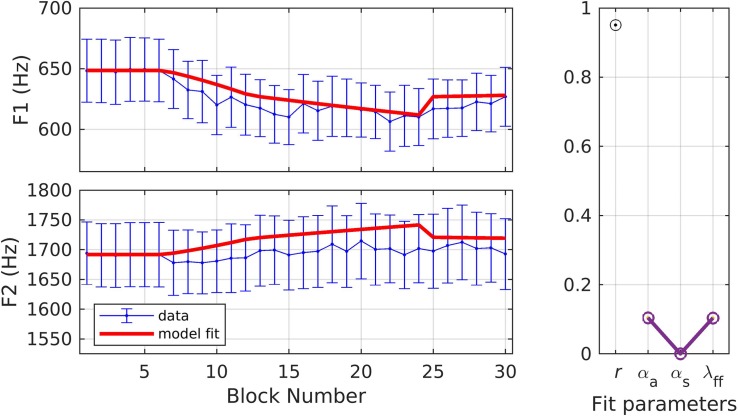
Simulation 3: model fits of a dataset with perturbations applied to both F1 and F2; F1 and F2 data are fit simultaneously (data from Daliri et al., 2018). **(Left)** Mean and standard error of experimental data in blue; model fit in red. **(Right)** Fit quality and optimized parameter values (*r* = correlation coefficient; α_*A*_ = auditory feedback control gain, α_*s*_ = somatosensory feedback control gain, and λ_*FF*_ = learning rate).

In simulation 3, parameters were optimized to fit both the F1 and F2 data simultaneously with one set of model parameters for both auditory dimensions. The model fit (Figure 5) had an *r* of 0.95, and the model fit for every block fell within the standard error of the data. The optimized parameter values were α_*A*_ = 0.10, α_*s*_ = 0.00, and λ_*FF*_ = 0.10. While λ_*FF*_ is within the range of simulations 1 and 2, the relatively low values of α_*A*_ and α_*s*_ indicate, within the SimpleDIVA interpretation, smaller sensory feedback-based corrections that, in turn, lead to lower compensation in this experiment compared to the prior experiments.

**FIGURE 6:**
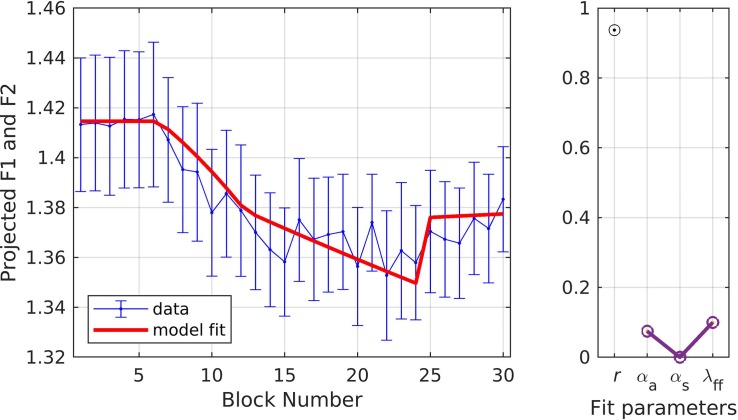
Simulation 4: model fits of a dataset with perturbations applied to both F1 and F2; F1 and F2 data are projected to a single vector (data from Daliri et al., 2018). **(Left)** Mean and standard error of experimental data in blue; model fit in red. **(Right)** Fit quality and optimized parameter values (*r* = correlation coefficient; α_*A*_ = auditory feedback control gain, α_*s*_ = somatosensory feedback control gain, and λ_*FF*_ = learning rate).

In simulation 4, the formant data were first normalized by dividing by the baseline average, then projected into a single dimension corresponding to the direction in F1/F2 space produced by the perturbation. This means that only the component of compensatory changes in F1/F2 that directly counteracted the perturbation were considered; this is similar to simulations 1 and 2, which only considered changes in F1 (the perturbed dimension) and ignored any changes in F2 that may also have occurred. The results for simulation 4, illustrated in Figure 6, are very similar to those of simulation 3 (*r* = 0.94;α_*A*_ = 0.07, α_*s*_ = 0.00, λ_*FF*_ = 0.10; model fit within the standard error of the data for every block), suggesting that projection of the results into a single dimension aligned with the perturbation is unnecessary as it produces essentially the same fit as fitting both the F1 and F2 datasets with a single set of parameter values.

### Simulation 5: Upward and Downward Perturbations of f_o_

Simulation 5 involves a dataset in which all participants underwent the adaptation paradigm under two counterbalanced conditions: one involving an upward shift in *f*_o_ and one involving a downward shift in *f*_o_ (Abur et al., 2018; data from only the healthy controls included here). Healthy older speakers of American English (*N* = 19, mean age = 65.3 ± 4.6 years) were instructed to vocalize a sustained/a/for three seconds while the stimulus appeared on a computer monitor. Both the *shift-up* and *shift-down* conditions followed a standard adaptation paradigm: 20 baseline, 60 ramp, 40 hold, and 40 after-effect trials. During the shift-up condition, *f*_o_ was increased by 1.69 cents for each ramp trial, reaching a maximum perturbation of 100 cents (a cent is a logarithmic unit of measure of changes in frequency, where 100 cents = 1 semitone). During the shift-down condition, the perturbation was applied in the same manner in the opposite direction reaching a maximum perturbation of −100 cents by the end of the ramp phase. Mean *f*_o_ was calculated for the duration of each 3-s trial using an autocorrelation method in Praat software (Boersma, 2001). The mean *f*_o_ across every block of three trials was estimated and the blocked data were used for model fitting. On average, participants compensated 83.8 and 86.7% in the shift-up and shift-down conditions, respectively.

**FIGURE 7:**
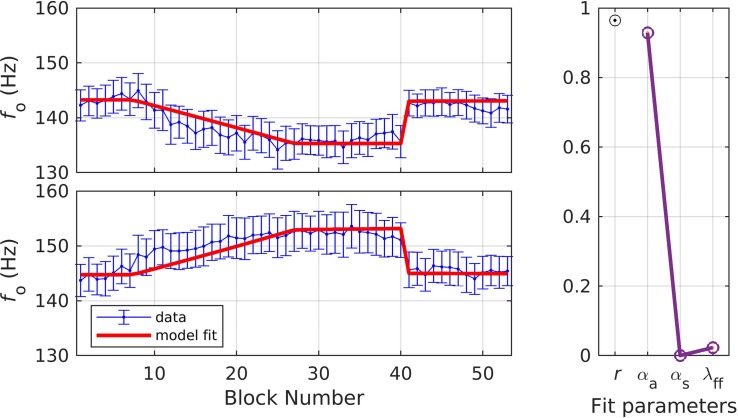
Simulation 5: model fits of a dataset with perturbations applied to fundamental frequency in both shift-up and down directions; shift-up and down data are fit simultaneously (data from Abur et al., 2018). **(Left)** Mean and standard error of experimental data in blue; model fit in red. **(Right)** Fit quality and optimized parameter values (*r* = correlation coefficient; α_*A*_ = auditory feedback control gain, α_*s*_ = somatosensory feedback control gain, and λ_*FF*_ = learning rate).

In simulation 5, a single set of parameters was used to fit both the shift-up and shift-down data simultaneously, as in simulation 3. The resulting fit fell within the standard error of the data in 96.2% of the experimental blocks (shown in Figure 7). The quality of fit and optimized parameter values were: *r* = 0.96, α_*A*_ = 0.93, α_*s*_ = 0.00, and λ_*FF*_ = 0.02. This simulation resulted in much higher values of α_*A*_ than prior simulations. Within the SimpleDIVA interpretation, a higher α_*A*_ is expected here since the long analysis window allowed for an unnaturally long amount of time for speakers’ auditory feedback correction to compensate for the perturbation. However, the very low value of α_*s*_ in these simulations was not expected; see section “Discussion” for further treatment. The next two simulations directly tested the effect of varying the analysis window on model parameters.

### Simulations 6 and 7: Late Versus Early Measurements of Perturbed f_o_

Similar to the previous dataset, the dataset modeled in simulations 6 and 7 involved an *f*_o_ perturbation experiment (Heller Murray, 2019; Heller Murray and Stepp, under review). The key feature of this dataset was that *f*_o_ was measured during two time periods – *early* and *late* in vocalization. Twenty young healthy speakers of American English (mean age = 21.0 ± 2.29) were asked to vocalize a sustained/a/for 3 s while the stimulus appeared on screen. They completed the task under three conditions: shift-up, shift-down, and control. The shift-up and down conditions followed the standard paradigm and each included a total of 60 trials: 15 baseline, 15 ramp, 15 hold, and 15 after-effect trials. No blocking of trials was performed due to the small number of total trials in the experiment. The ramp phase was characterized by a gradual change from 0 to a maximum perturbation of 100 cents (+ 100 cents in the shift-up condition and −cents in the shift-down condition). The control condition included a total of 60 trials without any perturbation and was used to account for the natural drift that occurs in *f*_o_ over time in the shift-up and down conditions. Median *f*_o_ was calculated using Praat software and custom MATLAB scripts, and each participant’s shift conditions were divided by their control condition to normalize the values. The two analysis time periods were: (1) between 20 and 120 ms after voicing onset (early); and (2) between 200 and 1500 ms after voicing onset (late). Note that in the early time window, feedback control will have had little time to “kick in” and thus lower values of α_*A*_ and α_*s*_ are expected compared to the later time window. The early time window also allows examination of model behavior in the near-absence of auditory feedback control (see Figure 2). When measured at the early timepoint, participants showed 19.1% (up-shift) and 50.9% (down-shift) compensation. When measured at the late timepoint, participants showed 29.8% (up-shift) and 51.5% (down-shift) compensation.

**FIGURE 8:**
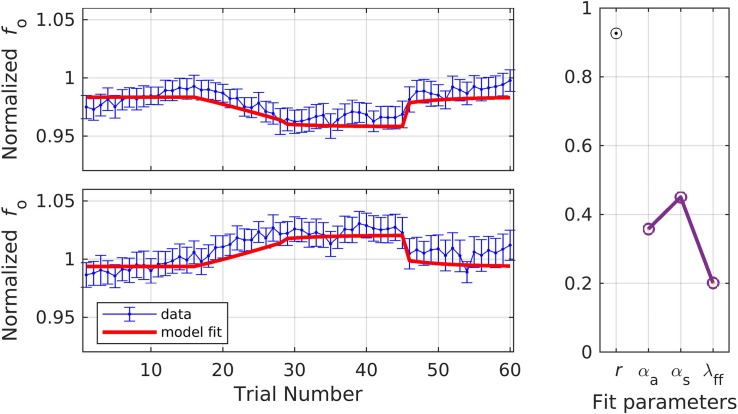
Simulation 6: model fits of a dataset with perturbations applied to fundamental frequency in both shift-up and down directions [normalized by an unshifted control condition; data from Heller Murray (2019)]. Shift-up and down data are fit simultaneously. Measurement of fundamental frequency was taken late in the trial (200–1500 ms after voicing onset). **(Left)** Mean and standard error of experimental data in blue; model fit in red. **(Right)** Fit quality and optimized parameter values (*r* = correlation coefficient; α_*A*_ = auditory feedback control gain, α_*s*_ = somatosensory feedback control gain, and λ_*FF*_ = learning rate).

In simulation 6, the model was fit to data measured at the late timepoint, which is in keeping with the model’s assumption that auditory feedback control has had a chance to contribute by the time the acoustic measurement is taken (i.e., that measurements occur 150 ms or more after perturbation onset). As before, a single set of parameters was used to fit both the shift-up and shift-down data simultaneously, with the resulting fits shown in Figure 8. The model fit fell within the standard error of the data on 68.3% of trials across both directions, and the resulting estimates were: *r* = 0.93, α_*A*_ = 0.36, α_*s*_ = 0.45, and λ_*FF*_ = 0.20.

**FIGURE 9:**
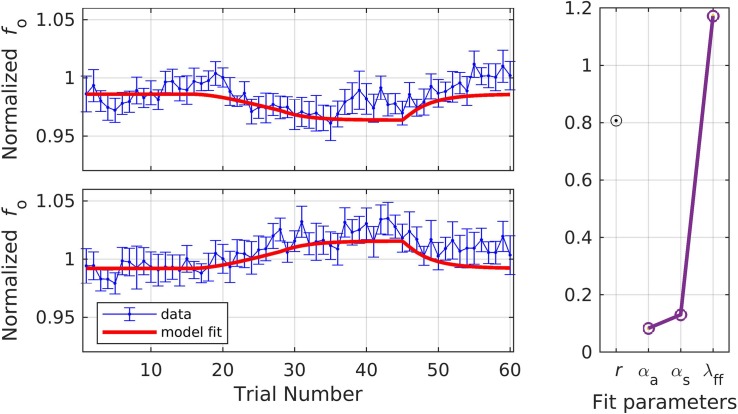
Simulation 7: model fits of a dataset with perturbations applied to fundamental frequency in both shift-up and down directions [normalized by an unshifted control condition; data from Heller Murray (2019)]. Shift-up and down data are fit simultaneously. Measurement of fundamental frequency was taken early in the trial (20–120 ms after voicing onset). **(Left)** Mean and standard error of experimental data in blue; model fit in red. **(Right)** Fit quality and optimized parameter values (*r* = correlation coefficient; α_*A*_ = auditory feedback control gain, α_*s*_ = somatosensory feedback control gain, and λ_*FF*_ = learning rate).

In simulation 7, the model was fit to data measured at the early timepoint (Figure 9), in violation of its implicit assumption of a measurement 150 ms or more after perturbation onset. For this simulation, we allowed the parameter λ_*FF*_ to go above 1 in order to achieve the optimal fit. The model still gives a reasonably good fit, though significantly poorer than in simulation 6, falling within the standard error of the data on 63.3% of trials. The overall quality of the fit and the optimized model parameters were: *r* = 0.81, α_*A*_ = 0.08, α_*s*_ = 0.13, and λ_*FF*_ = 1.17. Simulation 7 resulted in relatively low α values, which were expected within the SimpleDIVA interpretation due to the limited time for feedback control mechanisms to contribute to the production. This pattern likely resulted because the dataset violated the model’s assumption that feedback control mechanisms have kicked in by the time *f*_o_ is measured; the early time window used in simulation 7 results in unrealistically low α values and a small feedback-based correction according to Eq. 2, which in turn requires an unrealistically high value of λ_*FF*_in Eq. 3 to account for trial-to-trial changes.

### Simulations 8 and 9: Model Parameters From a Gradual Onset Perturbation Fit to a Sudden Onset Perturbation

The following simulations provide fits to data from an F1 experiment conducted under two counterbalanced conditions: one involving a gradual ramp phase (*gradual*) and one involving no ramp phase (*sudden*) (Chao and Daliri, unpublished data; see Supplementary Material for detailed methods). Fifteen young healthy speakers of American English (mean age: 21.7 ± 4.09) were instructed to produce the words “heck,” “head,” and “hep” with a word duration of 400–600 ms and loudness intensity of 72–82 dB SPL. Both conditions had a total of 180 trials with a maximum perturbation of 30% in F1. The gradual condition followed the standard paradigm, with 45 baseline, 45 ramp, 45 hold, and 45 after-effect trials. F1 was linearly increased during the ramp phase up to the maximum perturbation. The sudden condition had 45 baseline, 90 hold, and 45 after-effect trials. The maximum perturbation was introduced on the first trial of the hold phase. F1 trajectories were extracted using Audapter (Cai et al., 2008), which tracks formants based on linear predictive coding and dynamic programing. The average F1 was estimated in a window placed on the center of the vowel (40–60% of the vowel duration). Blocked data (mean of every three trials) were used for model fitting. Average compensation was 24.2% for the gradual condition and 23.7% for the sudden condition.

**FIGURE 10:**
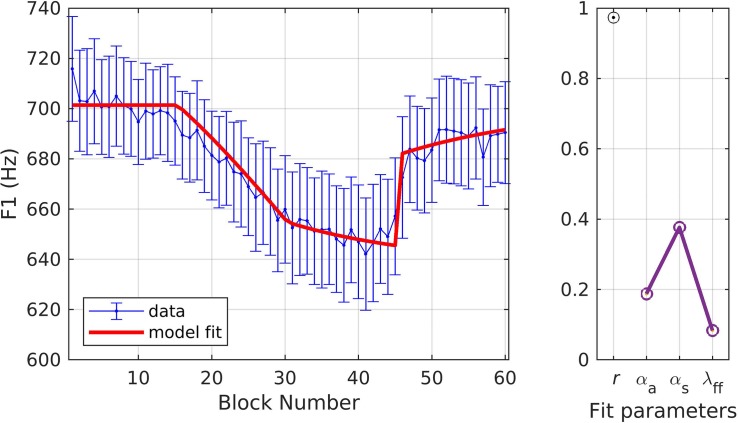
Simulation 8: model fits of a dataset with perturbations applied to F1 and with a gradual ramp phase (data from Chao and Daliri, unpublished data). **(Left)** Mean and standard error of experimental data in blue; model fit in red. **(Right)** Fit quality and optimized parameter values (*r* = correlation coefficient; α_*A*_ = auditory feedback control gain, α_*s*_ = somatosensory feedback control gain, and λ_*FF*_ = learning rate).

**FIGURE 11:**
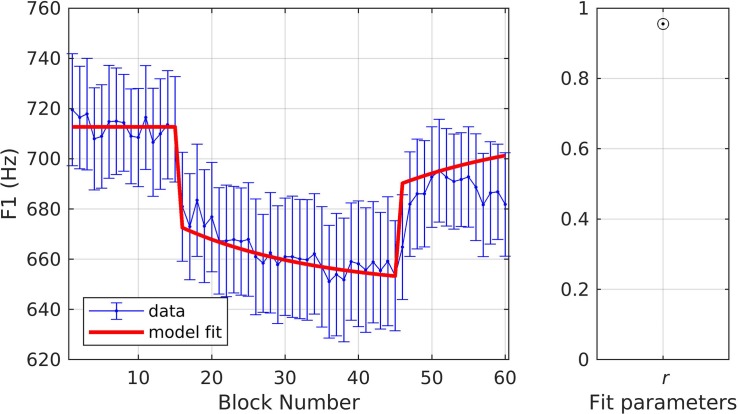
Simulation 9: model fits of a dataset with perturbations applied to F1 and with a sudden ramp phase. Model parameters were fixed using the parameters in simulation 9 (data from Chao and Daliri, unpublished data). **(Left)** Mean and standard error of experimental data in blue; model fit in red. **(Right)** Fit quality (*r* = correlation coefficient).

For these simulations, the goal was to first fit the model to one of the experimental conditions and then to use the resulting parameters to model the second condition, thus assessing how well the model could predict responses for a given experimental variation. In simulation 8, the model was fit to data from the gradual condition. The model fit fell within the standard error of the experimental data on all trials (Figure 10) and the quality of fit and optimized model parameters were: *r* = 0.97, α_*A*_ = 0.19, α_*s*_ = 0.38, and λ_*FF*_ = 0.08. These parameter values were then used to fit the data from the sudden condition (rather than finding optimal parameters for this condition). With α_*A*_, α_*s*_, and λ_*FF*_ fixed, the simulation predicted the same participants’ response to a variation of the adaptation paradigm (i.e., with no ramp phase). Figure 11 shows the resulting fits to the experimental data; the model fit is within the standard error on 98.3% of trials and estimates of fit quality indicated an excellent overall fit (*r* = 0.96).

The opposite was also true when the model was first fit to the sudden data (*r* = 0.97, α_*A*_ = 0.16, α_*s*_ = 0.21, and λ_*FF*_ = 0.07) and the resulting model parameters were used to fit the gradual data (*r* = 0.95). Together, these simulations highlight a strong predictive ability of the model across experimental conditions employing different patterns of perturbation.

### Simulation 10: Identifying Representative Parameter Values Across F1 Adaptation Studies

**FIGURE 12:**
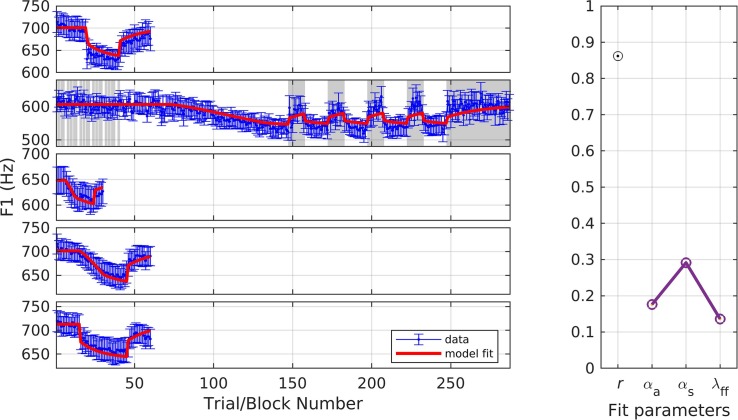
Simulation 10: model fits across all included F1 adaptation studies. From top to bottom: data from Haenchen (2017), Ballard et al. (2018), Daliri et al. (2018), and Chao and Daliri (gradual and sudden conditions; unpublished data). Noise masked trials indicated with gray shading. **(Left)** Mean and standard error of experimental data in blue; model fit in red. **(Right)** Fit quality and optimized parameter values (*r* = correlation coefficient; α_*A*_ = auditory feedback control gain, α_*s*_ = somatosensory feedback control gain, and λ_*FF*_ = learning rate).

In the final simulation, we fit F1 data from all of the formant studies described above (simulations 1, 2, 3, 8, 9) using a single set of parameters. Figure 12 shows the resulting fits. The fit quality and optimized parameter values were: *r* = 0.86, α_*A*_ = 0.18, α_*s*_ = 0.29, and λ_*FF*_ = 0.14. These model estimates provide representative values that can be used to predict responses in future formant adaptation studies.

To assess the possibility that these representative parameter values are overfitting our particular datasets, we performed a leave-one-out cross-validation procedure in which the model was fit to four of the five datasets, with the optimized parameters then used to fit the fifth (test) dataset (repeated five times, with each dataset acting as the test set once). The average *r* for the test set in these five simulations was 0.91, indicating that the model’s fit quality extends beyond datasets used in the optimization procedure^4^. The parameter ranges obtained across the five simulations were 0.17–0.20 for α_*A*_, 0.25–0.32 for α_*s*_, and 0.11–0.15 for λ_*FF*_.

To further assess the reliability of these parameters, we utilized a percentile bootstrap estimation procedure (Efron and Tibshirani, 1993) to obtain 95% confidence intervals for each parameter. 1000 iterations were performed, with the data for each iteration formed as follows. For each of the five studies, a new dataset was formed by sampling subjects with replacement from the original dataset, and the average of these data was calculated. Then SimpleDIVA was used to simultaneously fit these five averages using a single set of parameters. This resulted in a distribution of 1000 estimates for each parameter, from which the 95% confidence interval was drawn. The resulting confidence intervals were 0.13–0.21 for α_*A*_, 0.17–0.38 for α_*s*_, and 0.06–0.38 for λ_*FF*_.

## Discussion

The aim of this article was to describe and test a simple 3-parameter model, SimpleDIVA, that can disentangle the roles of auditory feedback, somatosensory feedback, and feedforward control processes during sensorimotor adaptation experiments. We tested the model using six existing datasets collected in different laboratories and with numerous variations in the sensorimotor adaptation paradigm. The model provided close fits to data from these studies, which spanned experiments: of formant and pitch perturbations; with/without masking-noise trials; with perturbations in single and multiple auditory dimensions; with measurements made in different analysis windows of the acoustic signal; and when predicting model fits from one experimental condition to another. The model simulations highlighted the effectiveness of the model in estimating the relative contribution of feedback and feedforward control systems to sensorimotor learning and providing excellent fits to the data, with a mean Pearson’s *r* of 0.95 ± 0.02 across the studies modeled here (excluding simulation 6 that was included to illustrate the effect of analysis time window). In addition, the simulations revealed properties of the model (and of sensorimotor adaptation) that we will discuss in detail below.

### Role of Somatosensory Feedback in the Absence of Auditory Feedback

Previous studies have used noise-masked trials as a method of assessing sensorimotor adaptation in the absence of auditory feedback (e.g., Houde and Jordan, 1998; Ballard et al., 2018). A residual compensatory effect is observed in noise-masked trials during the hold phase, indicative of adapted feedforward commands. However, prior studies typically did not consider the effects of somatosensory feedback control during noise-masked trials (but see discussion in Ballard et al., 2018). In simulation 2, the SimpleDIVA model was fit to the data from one such study and revealed an interesting and somewhat unintuitive finding: when producing speech under masking noise in the hold phase, participants show gradual *de-*adaptation despite the fact that there is no auditory signal available. This aspect of the data is captured by the model since masking noise does not eliminate somatosensory feedback, and thus the somatosensory feedback controller is attempting to move the vocal tract back toward its pre-perturbation configuration; the resulting corrective movements generated by the somatosensory feedback controller lead to updating of the feedforward commands, in turn resulting in the de-adaptation evident in the experimental data and model fit. Thus, the model highlights a previously ignored aspect of speech sensorimotor adaptation experiments that involved masking noise during the hold phase, while at the same time providing an explanation for this phenomenon. Notably, this effect is analogous to findings in the visuomotor literature showing de-adaptation toward baseline in the absence of visual feedback (Hay et al., 1965; Scheidt et al., 2005; Smeets et al., 2006).

### Optimized Model Parameters Change as a Function of Experimental Protocol Variation

Although the optimized parameters were often similar across simulations, differences were observed that are likely at least partially due to differences in experimental design. For example, the model was sensitive to differences in the period of signal selected for analysis. Simulations 6 and 7 demonstrated the effect of varying the measurement window directly. In simulation 6 an early time window of 20–120 ms after voice onset was used, thus minimizing the contribution of feedback control mechanisms, which do not start affecting movement until approximately 50 ms after perturbation detection for somatosensory feedback control and over 100 ms after perturbation detection for auditory feedback control (see Burnett et al., 1998; Guenther, 2016). As expected, this resulted in much lower feedback control gains in the optimal model fit (α_*A*_ = 0.09, α_*s*_ = 0.15) compared to simulation 7, which used a later time window of 200–1500 ms after voice onset and obtained optimized values of α_*A*_ = 0.39 and α_*s*_ = 0.44.

In an *f*_o_ perturbation experiment that had a very long measurement window (∼3 s), the model estimated that sensorimotor control was dominated by the auditory feedback control system, with α_*A*_ = 0.93 (simulation 5). Although a high gain for α_*A*_ is expected due to the measurement window extending so long beyond perturbation onset, these simulations identified no contribution of the somatosensory feedback controller (i.e., α_*s*_ = 0.00) rather than a higher than normal contribution that might be expected due to the long analysis time window. This unexpected finding indicates that, unlike the formant perturbation studies involving shorter/earlier time windows simulated herein where adaptation plateaus at approximately 25–50% of the perturbation size, adaptation in the *f*_o_ perturbation study of Abur et al. (2018) was nearly complete (85.3%); in terms of the model, this is because somatosensory feedback control mechanisms are not acting to limit the amount of compensation. This finding may reflect a situation in which auditory feedback control dominates due to the use of unnaturally long (3 s) steady state vowel productions, which may have allowed participants to consciously “pitch match” their production to the target pitch, thereby overcoming the natural tendency for somatosensory feedback to limit the amount of compensation. Further study is needed to verify or refute this interpretation.

Further experimental choices that could affect model parameters include the loudness of the auditory feedback signal (with a louder signal possibly resulting in more auditory error detection and within-trial correction, evidenced by a larger α_*A*_), the use of low levels of masking noise in combination with normal and perturbed auditory feedback (possibly lowering the amount of error detection and correction, evidenced by a smaller α_*A*_), or the use of anesthesia on the speech articulators (which should lead to a decrease in α_*s*_ and a concomitant increase in overall compensation to an auditory perturbation). Future studies will investigate these possibilities.

### Relationships Between Somatosensory and Auditory Feedback Control Gains

An interesting finding in the simulations is that, in general, within-trial corrections based on somatosensory feedback seemed to be associated with the magnitude of compensation. That is, lower somatosensory feedback control gains occurred with lower auditory feedback control gains, and vice versa (simulation 5 was the exception). This result is not unexpected when one considers how the time window over which acoustic measurements are made affects the model parameter estimates: put simply, later time windows show evidence of more feedback control, both auditory and somatosensory.

In the model, increasing both auditory and somatosensory gains proportionally (e.g., going from α_*A*_ = 0.2, α_*s*_ = 0.4 to α_*A*_ = 0.3, α_*s*_ = 0.6) has no effect on the maximum amount of compensation that is achieved during a sufficiently long hold phase. To see why, note that the extent to which the auditory feedback control system opposes a perturbation directly affects the extent to which the somatosensory feedback control system will detect a mismatch from the normal configuration for the sound, in turn affecting the amount the somatosensory feedback control system opposes any corrective contributions from the auditory feedback control system. Ultimately, this competition between auditory and somatosensory feedback controllers determines the maximum compensation that can occur as a percentage of the perturbation size according to the following equation:

(4) $$M\,a\,x\,C\,o\,m\,p\,e\,n\,s\,a\,t\,i\,o\,n=\alpha_{A}/\left(\alpha_{A}+\alpha_{S}\right)$$

For example, if the auditory and somatosensory feedback gains are equal, the maximum compensation achieved by the model will be 0.5, or 50% of the perturbation size. This equation also helps explain why model fits to the data from Abur et al. (2018), which showed near-complete compensation, resulted in an optimized α_*s*_ of 0.00.

Although increasing α_*A*_ and α_*S*_ proportionally does not affect the maximum level of compensation, it does affect the amount of within-trial compensation seen for trials shortly after a perturbation is induced. This is because the feedback-based correction calculated in Eq. 2 will be larger if α_*A*_ and α_*S*_ are both larger. Furthermore, for a given value of λ_*FF*_, increasing α_*A*_ and α_*S*_will lead to faster adaptation of the feedforward command according to Eq. 3.

Notably, if the *ratio* of α_*A*_ to α_*S*_ changes (as opposed to both of them increasing/decreasing proportionally), then we expect more adaptation (for greater α_*A*_/α_*S*_ratios) or less adaptation (for smaller ratios) after many training trials. Indeed, it is the ratio of these parameters that determines the degree of maximal compensation that will occur in the model since it captures the essence of the competition between the auditory and somatosensory feedback controllers discussed above. Again, different experimental paradigms may lead to somewhat different α_*A*_/α_*S*_ ratios, in part because the delays in the two feedback control systems are different, which in turn means the relative influence of α_*A*_ compared to α_*S*_ depends on the point in time the acoustic measurement for the trial is made (see Figure 2 and associated text). Following findings of individual preferences for auditory or somatosensory feedback control reported in some prior studies (e.g., Lametti et al., 2012), it is likely that the ratio of α_*A*_ to α_*S*_ also differs considerably across individuals.

In sum, the relative values of α_*A*_ and α_*S*_ determine the maximum amount of compensation that can occur in the model, whereas the absolute values of α_*A*_ and α_*S*_ affect the rate at which the model converges to this maximum compensation level during the hold phase.

### Predictive Power of SimpleDIVA

To test the predictive power of the model, we identified optimal model parameters from data in one experimental condition involving a gradual perturbation onset and applied the parameters to a second experimental condition in which the perturbation onset was abrupt (simulations 8/9). The quality of the predicted fit was excellent (correlation coefficient of 0.96) and fell within the range of the other simulations in this article. Not only can SimpleDIVA provide an insight into the mechanisms underlying sensorimotor adaptation, but the model can also predict responses for an experiment using data from a prior experiment.

In the final simulation (simulation 10), we fit the model simultaneously across five F1 datasets with variations in the experimental design. The resulting parameters provide a reference point for expected model parameters in F1 adaptation studies and may be used to predict responses in future studies. Using the model in this way supports the development of clear hypotheses that can be tested empirically to ultimately advance the field of speech motor control.

### Limitations of the Model

In this article, we have demonstrated how SimpleDIVA can be used across a number of different adaptation paradigms. One experimental variation that is not currently supported by SimpleDIVA is the setting of individually-derived perturbation magnitudes (e.g., a 20% shift in an individual’s F1/F2 space toward another vowel; Schuerman et al., 2017). In future iterations, we plan to make it possible to specify the perturbation magnitude at the level of the individual, rather than only at the group level. The model is also not yet designed to address the results of studies involving unexpected perturbations rather than the sustained perturbations used in the studies covered herein.

One important limitation of the model for fitting sensorimotor adaptation data concerns the assumption that feedback control mechanisms have started to contribute by the time that the acoustic measurement is taken, ideally at least 150 ms after perturbation onset. Most prior studies of sensorimotor adaptation encourage participants to lengthen their vowel productions in order to increase the amount of adaptation under perturbed feedback, making the data amenable to fitting by SimpleDIVA. However, the typical durations of some vowels during normally produced sentences are less than 150 ms (Jacewicz et al., 2007). For single-syllable stimuli with these vowels, it is unlikely that auditory feedback control substantially affects within-trial performance, though somatosensory feedback control mechanisms are likely contributing. The model’s applicability to such cases is thus questionable.

A potential issue involving non-uniqueness of solutions can arise in the current version of the model when one of the model parameters assumes a value that is very close to zero. For example, if the optimal solution involves a value of λ_*FF*_ equal to zero, the value of α_*S*_ no longer has an effect on the fit quality, and the model’s optimization routine may find a different value of α_*S*_ each time it is run despite achieving the same fit quality each time. This is not a shortcoming of the model *per se*; rather, it is an indication that the solution space is non-unique in these cases, with many possible solutions (typically an infinite number) providing the same optimal fit. This behavior is not likely to occur for neurologically normal participant groups (for whom the model parameters should not approach zero) but could possibly occur in certain disordered participant groups or when the individual trials of the perturbation experiment involve unusually long, drawn out perturbed utterances as described above with respect to simulation 5.

Another potential limitation of the model concerns an inherent assumption that the relative contributions of the auditory and somatosensory feedback controllers to adaptation of the feedforward command is the same as their relative contributions to online, within-trial corrections. This is because only a single adaptation rate parameter (λ_*FF*_) is used, rather than separate rates for auditory and somatosensory feedback contributions. This assumption has not yet been experimentally verified; if it proves to be false, the model may need to be modified to include separate adaptation rates for auditory and somatosensory error-based updates of the feedforward command.

Another potential limitation of the model is the inclusion of only one form of learning: adaptation of feedforward motor programs. The model can be extended to allow other forms of learning, such as changing of the auditory and/or somatosensory targets for a speech sound. Changes to these targets are expected to occur on a much slower time scale – longer than the time scale of a single adaptation experiment – according to the model (see Guenther, 2016 for details). For example, targets may change over the course of speech development in children or over a longer period of speech therapy for those with communication disorders. Some studies have shown changes to perceptual category boundaries for speech sounds after speech motor learning (e.g., Shiller et al., 2009). Although this might be construed as evidence for changes to the production target for the speech sound over the course of an experimental session, this interpretation is tenuous since (i) the link between *perceptual* category boundaries and the targets for speech *production* remains unknown, and (ii) the production targets represent idealized versions of speech sounds, whereas adaptation effects on perception involve ambiguous stimuli at category boundaries. We performed simulations of versions of SimpleDIVA that included adaptation terms for auditory and/or somatosensory targets. If λ_*FF*_ is set to 0 and only sensory targets are allowed to adapt, the model’s fits are poorer than for the version described here. If the sensory targets are allowed to adapt while still includingλ_*FF*_, model fits showed almost no improvement over the simpler version included here, and solutions were often non-unique. For these reasons sensory target adaptation was omitted from the simulations included in this article.

Finally, the simulations herein have focused on fits to group average data. The cross-validation and bootstrap confidence interval estimation analyses performed as part of simulation 10 indicate reliable ranges for each parameter when fitting F1 perturbation group datasets (N of 10 or more for each of the five studies analyzed here). They do not address questions regarding parameter stability within a single study, such as how many subjects are necessary in a group to obtain stable parameter values (a complex topic beyond the scope of the current article). Thus, significant caution is warranted when interpreting differences in parameter values across studies; the interpretations presented here are based on the model’s theoretical foundations rather than direct statistical comparisons.

### Future Directions

The current set of simulations focused on modeling data primarily from young healthy adult speakers (only simulation 5 included data from older adults). A key next step will be to expand this work to examine the contribution of feedback and feedforward control to sensorimotor learning across the lifespan and in those living with communication disorders. Model parameter values derived from multiple participant groups, for example, a neurotypical group and a group with a disorder, can be compared to illuminate the between-group differences in speech motor processing. This line of research has the potential to identify underlying mechanisms of communication disorders with a sensorimotor basis and to subsequently pave the way for the development of future treatments. An important step in the model development process for this purpose will be the creation of statistical tests of the reliability of parameter value differences between participant groups.

Another important future direction is to investigate the model’s capabilities for reliably characterizing speech motor control processes in individuals. The current simulations were all fits to group average data, which does not capture individual variation in the relative use of auditory feedback, somatosensory feedback, and feedforward control processes. Previous studies of adaptive responses have shown increased variation among disordered populations (e.g., Abur et al., 2018) as well as individual preferences for one sensory modality over another (Lametti et al., 2012). Future studies examining parameters derived from individual subjects will be necessary to assess how robust the model estimates are at the level of an individual, including individuals with speech motor disorders. Specific issues of importance are whether individual subjects can be fit reliably from a single experimental session and the degree to which the fits are unique and stable (e.g., could a near-optimal fit be achieved with wildly different parameter values even though the optimal fit is unique?).

A third future direction concerns testing of model predictions in order to better verify its assumptions. Unfortunately, direct verification through physiological, structural, or behavioral measures is not possible. One reason for this is that, in terms of physical aspects of the brain, the model parameters correspond to rather large-scale and difficult (if not impossible) to measure characteristics such as number of synaptic projections between areas, strengths of these synapses, plasticity of these synapses, and neural sensitivity of the auditory and somatosensory periphery. Model predictions regarding the relationship between adaptation and online corrections can be tested, but it is noteworthy that even the within-trial, online response to an auditory perturbation depends on factors other than the auditory feedback control gain since these within-trial responses are, like adaptive responses, also dependent on feedforward and somatosensory feedback control mechanisms. For this reason, we are currently formulating a version of SimpleDIVA that is aimed at within-trial responses to unexpected perturbations. This requires the addition of parameters representing the temporal delays in the auditory and somatosensory feedback control loops, which are not considered in the current version of the model. Adding these new parameters presents challenges regarding finding unique fits that we are currently addressing. Upon completion of this version of the model, it should be possible to test the model’s ability to account for within-trial time courses as well as adaptation over many trials within the same subject. However, this topic is beyond the scope of the current manuscript, which has a primary aim of demonstrating how a simple model characterizing the three main motor control processes in speech can provide excellent fits to a wide range of auditory sensorimotor adaptation data.

Finally, SimpleDIVA is not the only computational model used to examine sensorimotor adaptation. For example, state space models have been widely used in studies of limb motor adaptation (Thoroughman and Shadmehr, 2000; Smith et al., 2006; Galea et al., 2015; Huberdeau et al., 2015) and such a model was recently applied to speech (Daliri and Dittman, 2019). While the state space model provides good fits to speech sensorimotor adaptation data, it is limited by the fact that the two model parameters (an internal estimate forgetting factor and a sensory error weighting factor) cannot differentiate auditory and somatosensory feedback control processes from feedforward control processes. SimpleDIVA’s third parameter (compared to only two for the state space model) gives it this ability without adversely affecting the model’s ability to find a unique optimal solution. Furthermore, the adaptation process captured by SimpleDIVA is, in essence, the same process that is used in the full DIVA model to develop accurately tuned speech motor programs in the first place; no such connection exists for state space model parameters. Further treatment of the relatively advantages and disadvantages of SimpleDIVA and state space modeling approaches is beyond the scope of the current article; we plan to address this important topic in a future study.

## Data Availability Statement

The datasets generated for this study are available on request to the corresponding author.

## Ethics Statement

This study was carried out in accordance with the recommendations of the Institutional Review Board at Arizona State University with written informed consent from all subjects. All subjects gave written informed consent in accordance with the Declaration of Helsinki. The protocol was approved by the Institutional Review Board at Arizona State University.

## Author Contributions

FG contributed conception and design of the study. FG, AN-C, RF, EK, and HW developed and programed the SimpleDIVA model. AD, DA, KB, S-EC, S-CC, EH, and TS collected and processed data. EK and HW tested model simulations. EK wrote the first draft of the manuscript. AD wrote the Supplementary Material. All authors contributed to manuscript revision, read, and approved the submitted version.

## Conflict of Interest

The authors declare that the research was conducted in the absence of any commercial or financial relationships that could be construed as a potential conflict of interest.

## Footnotes

1 Note that the names used to describe the phases are not always the same as those used in this article. We will use the terms defined here throughout the article to avoid confusion.

2 For example, it does not make sense within the model for the auditory feedback gain to be less than 0 (which would exacerbate rather than correct auditory errors) or greater than 1 (which would overcompensate for auditory errors).

3 The model will still typically identify an auditory feedback gain that is greater than zero because such a gain is needed to account for trial-to-trial changes in the acoustic parameter being perturbed (see Eq. 3).

4 The careful reader might note that this cross-validated *r* value is actually higher than when all five datasets are used for fitting. This is possible because the optimization procedure minimizes RMSE as described in Section “Materials and Methods” rather than maximizing *r* directly.
