# Supplementary material for: A Simple 3-Parameter Model for Examining Adaptation in Speech and Voice Production
Source: Front Psychol. 2020 Jan 21;10:2995. doi: 10.3389/fpsyg.2019.02995 (PMC6985569; doi:10.3389/fpsyg.2019.02995)
Supplement: Supplementary file 1 [file Data_Sheet_1.PDF]

Simulations 8 and 9: Experimental Data Methods

The dataset modeled in Simulations 8 and 9 has not previously been published. The data collection and processing methods are detailed here for completeness.

1 Participants

We recruited 15 healthy native speakers of American English (12 female participants; mean age = 21.736; SD age = 4.086; age range 18.91-35.79 years) for this study. Participants were recruited from participant pool of undergraduate students of Arizona State University. Prior to the experiment, participants were screened to exclude those with history of neurological, psychological, speech-language disorders, and hearing disorders (pure tone hearing threshold ≤20 dB HL at octave frequencies from 250 to 8000 Hz). Written informed consent was obtained from all participants before start of the experiment, and all study protocols were approved by the Institutional Review Board at Arizona State University.

2 Procedure

The experiment was conducted inside a sound attenuated booth. A microphone (SM58, Shure) was placed ~15 cm from the corner of the participant's mouth. The microphone signal was amplified (Tubeopto 8, ART) and passed to computer via an audio interface (Ultralite Mk3 hybrid, MOTU). We used Audapter with Auditory Stream Input output (ASIO) driver to access and process the amplified microphone signal and feed back to the participant in in real-time (input-output lag < 18 ms) (Cai, 2015). Output signals were amplified (Pro Rx1602, Eurorack) and played back to the participant via insert earphones (ER-1, Etymotic Research Inc.). Prior to each experiment, we calibrated the input-output level so that the played-back signal was 5 dB greater than the microphone signal.

Before the start of the experiment, participants were instructed to read aloud as soon as the word appeared on the computer screen. Participants completed a set of 30 practice trials to become familiar with producing the target words with appropriate intensity and duration. Participants produced monosyllabic words with the structure h-vowel-consonant (hVC) ("heck" "head", and "hep"). Each trial lasted 2.5 s and then there was a blank screen that lasted for 1–2 s. During the practice trials, after each production, participants received visual feedback regarding their intensity and duration to ensure they produce words within the desired intensity (72–82 dB SPL) and duration ranges (400–600 ms). After the practice trials, participants completed two blocks of adaptation conditions (gradual and sudden perturbations). The order of blocks was randomized for participants (7 participants completed gradual adaptation first). We used a filler task (sentence reading task) between the two blocks of the adaptation trials to reduce the effect the two conditions on each other (30 randomly selected sentences from the Harvard Sentence list). Additionally, participants produced hVC words containing three front vowels ("hip", "hep", "hap") to further reduce the effect of conditions on each other. Each adaptation block consisted of 180 trials (60 repetitions of the "heck" "head", and "hep" with random order). In both adaptation conditions, the first 45 trials were baseline trials in which normal auditory feedback was provided to participants. In the second 45 trials of the gradual condition, auditory perturbation (increase in the first formant; F1) was gradually increased to reach 30% shift in F1. In the second 45 trials of the sudden condition, auditory perturbation (30% increase in F1) was applied. In the third 45 trials of both conditions, auditory perturbation (30% increase in F1) was applied. Finally, in the last 45

trials of both conditions, normal auditory feedback was provided to participants. For real-time formant tracking and manipulation, we used Audapter with default parameters. To enhance formant tracking of Audapter, we used a MATLAB (MathWorks Inc.) script to automatically extract average formant values of practice trials, and then, we used the participant-specific average formants to fine-tune Audapter. Formant tracking in Audapter is based on linear predictive coding (LPC) analysis and dynamic programming.

After the experiment, we first manually annotated the vowel onset and offset for all production based on the spectrograms of the signals. This process accounted for formant tracking errors made by Audapter and for mispronunciations done by participants and thus, we excluded these errors from data analysis (< 1% of all trials). Then, for each trial, we averaged the first and second formants (provided by Audapter) in a window placed on the center of the vowel (40–60 % into the duration of the vowel).
